# Accuracy and Reliability of Whole Blood Bilirubin Measurements Using a Roche Blood Gas Analyzer for Neonatal Hyperbilirubinemia Screening and Risk Stratification

**DOI:** 10.3389/fped.2022.910566

**Published:** 2022-07-04

**Authors:** Qing Wang, Tianyi Zhang, Yuanxi Lin, Li Jiang, Wenlong Zhou, Xiaolong Zong

**Affiliations:** ^1^Department of Clinical Laboratory, The General Hospital of Tianjin Medical University, Tianjin, China; ^2^Department of Emergency Medicine, The Second Hospital of Tianjin Medical University, Tianjin, China; ^3^College of Medical Laboratory Science, Tianjin Medical University, Tianjin, China; ^4^Department of Clinical Laboratory, The Second Hospital of Tianjin Medical University, Tianjin, China

**Keywords:** neonatal hyperbilirubinemia, bilirubin measurement, whole-blood bilirubin, accuracy, clinical reliability

## Abstract

**Background:**

Accurate bilirubin measurements are essential for appropriate management of neonatal hyperbilirubinemia. This study aimed to evaluate the accuracy and reliability of whole blood bilirubin measurements obtained using a Roche blood gas analyzer (Roche TBiL), with total serum bilirubin (TSB) measurements determined by the Ortho VITROS 4600 chemistry system (Ortho TSB) serving as a reference.

**Materials and Methods:**

Medical records of hospitalized neonates that underwent simultaneous Roche TBiL and Ortho TSB measurements were reviewed for eligibility selection and data collection. The correlations and differences between two sets of results were determined using Passing–Bablok regression analysis and a Bland–Altman plot, respectively. For eligible newborns, the risk of developing severe hyperbilirubinemia was assessed using the Bhutani nomogram. Weighted kappa analysis was used to evaluate the agreement between risk prediction by the two methods.

**Results:**

We obtained 618 paired Roche TBiL and Ortho TSB results from 309 neonates. Roche TBiL and Ortho TSB measurements showed a good correlation (*r* = 0.923; 95% CI: 0.905–0.938). Passing–Bablok regression analysis yielded the following equation: Roche TBiL = 0.794 × Ortho TSB + 1.255 mg/dL, with a slope of 0.794 (95% CI: 0.763–0.825) and intercept of 1.255 (95% CI: 1.042–1.417). The average difference between the two methods was 0.1 ± 1.448 mg/dL. A total of 207 neonates were eligible for evaluation of the agreement between the risk-grading methods. Although kappa analysis showed good agreement between the methods, with a weighted kappa of 0.681 (95% CI: 0.610–0.751) across all populations, the values for approximately half of the neonates at intermediate and high risk of hyperbilirubinemia (33/72) were underestimated by Roche TBiL.

**Conclusion:**

Our results indicate that Roche TBiL and Ortho TSB measurements in the neonatal population are not consistent. As a point-of-care and trace blood assay, Roche blood gas bilirubin measurements can facilitate primary screening of neonatal hyperbilirubinemia, but it seems to lack accuracy regarding risk stratification, particularly for high-risk newborn individuals.

## Introduction

Hyperbilirubinemia is among the most common causes of hospitalization in neonates. Although most forms of jaundice are physiological and self-limiting, severe cases will result in overwhelming bilirubin deposition in the brain without timely treatment, leading to irreversible neurological damage, namely, kernicterus (1, 2). Bilirubin measurements are fundamental for diagnosis, risk stratification, and treatment decision-making in cases of neonatal hyperbilirubinemia (3). The initiation of phototherapy for neonates with severe hyperbilirubinemia or at high risk of subsequent severe hyperbilirubinemia is commonly assessed using a combination of risk factors evaluation and the Bhutani’s hour-specific bilirubin nomogram, which was established on the basis of total serum bilirubin (TSB) measurements obtained by the diazo method (1, 2, 4).

In recent decades, the technology underlying bilirubin assays has evolved significantly. New methods such as trace sample-based blood gas bilirubin measurements performed by Co-oximetry now provide more choices and convenience for clinicians (3). Blood gas analyzers such as the Roche Cobas models support multi-test panels and deliver test results quickly; consequently, they are commonly used in neonatal intensive care units (NICUs). However, an important prerequisite for these blood gas analyzers is that their whole blood bilirubin measurements must be comparable to measurements obtained with TSB, which remains the gold standard (3). In the current study, we compared whole blood bilirubin measurements on a Roche blood gas analyzer (Roche TBiL; Roche Diagnostics GmbH, Germany) with TSB measurements obtained by the Ortho VITROS 4600 chemistry system (Ortho TSB; Ortho Clinical Diagnostics, United States) from 309 hospitalized neonates to evaluate the accuracy and clinical reliability of Roche TBiL measurements.

## Materials and Methods

### Study Population

Medical records of neonates hospitalized in the NICU of Tianjin Medical University General Hospital between April 2019 and June 2021 were reviewed for eligibility and data collection. Neonates who underwent simultaneous Roche TBiL and Ortho TSB measurements (determined according to the sampling time recorded in the Laboratory Information System) were included for the measurement accuracy study, and data on demographic characteristics, such as gestational age, age post-delivery, and bilirubin concentrations were collected. Term newborns aged between 12 and 144 h were identified as eligible for the further clinical reliability evaluation study. This study was approved by the Ethics Committee of the General Hospital of Tianjin Medical University (no. IRB2022-WZ-011).

### Quantitative Bilirubin Assay

For the whole blood bilirubin assay, 0.2 mL of blood collected into BD^®^ blood gas syringes containing balanced heparin was analyzed using a Roche Cobas b123 (Roche Diagnostics GmbH, Germany), a point-of-care automated blood gas analyzer. This analyzer measures total bilirubin and hemoglobin derivatives in hemolyzed whole blood samples by using direct spectrophotometry in the Co-oximetry module. Plasma-equivalent bilirubin measurements are then reported through a calculation that relies on the measurement of hemoglobin, which is converted to hematocrit to correct for the dilution effect of red blood cells. Because the Roche Cobas b123 utilizes spectrophotometry for quantitative measurements, it cannot distinguish the different fractions of bilirubin.

For TSB measurements, another 1-mL blood sample was collected into vacuum tubes, centrifuged for serum collection, and further analyzed using the multi-layered slide technique by reflectance spectrophotometry on a VITROS 4600 Chemistry analyzer (Ortho Clinical Diagnostics, United States). VITROS BuBc Slides were used for quantitative measurements of unconjugated bilirubin (Bu) and conjugated bilirubin (Bc) concentrations in neonatal serum and plasma, and the TSB concentration was calculated. The calibration of Bu was traceable to a standard reference material, NIST 916a, and Bc was calibrated using the synthetic di-tauro bilirubin disodium salt. During the study period, the actual variation in Bu and Bc measurements, assessed according to the internal quality control lot, ranged from 3 to 5% and 4 to 8%, respectively.

### Severe Hyperbilirubinemia Risk Evaluation

The risk of developing subsequent severe hyperbilirubinemia in eligible newborns was evaluated using the Bhutani nomogram (4), which ranks an infant’s risk according to their infant’s age in hours. High risk was defined as age-specific TSB levels that reached or exceeded the 95th percentile. Intermediate risk was subdivided into high- and low-intermediate risks, with the 75th percentile serving as the threshold. Low risk was defined as age-specific TSB levels below the 40th percentile.

### Statistical Analysis

Data showing a normal distribution were expressed as mean and standard deviation; data that did not show a normal distribution were expressed as median values with interquartile ranges. Correlation, Passing–Bablok regression, and Bland–Altman analyses were used to compare the two methods. The agreement between severe hyperbilirubinemia risk prediction by Roche TBiL and Ortho TSB was determined by weighted kappa analysis. All data analysis procedures were performed using MedCalc 15.2.2 software (MedCalc Software Ltd., Belgium).

## Results

**FIGURE 1:**
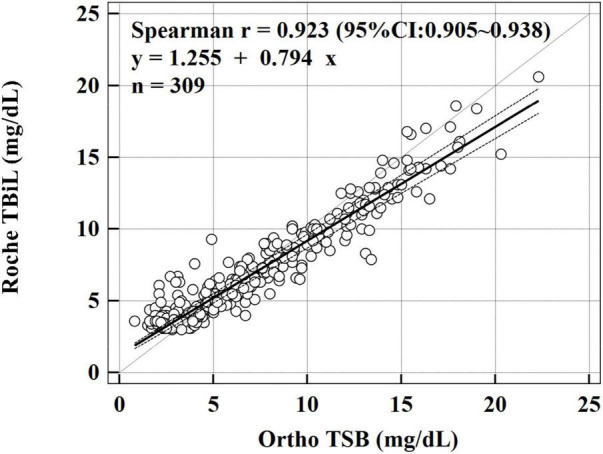
Passing–Bablok linear regression analysis between Roche TBiL and Ortho TSB. The solid line is the line of best fit, and the dashed lines reflect the 95% CI. The dotted line represents the line of equivalence. The regression equation is Roche TBiL = 0.794 × Ortho TSB + 1.255 mg/dL.

During the study period, 618 paired bilirubin measurements from 309 hospitalized neonates were available for analysis. The characteristics of the study population are shown in Table 1. We first explored the linear relationship between Roche TBiL and Ortho TSB by using Spearman’s correlation analysis. As shown in Figure 1, the two sets of values were closely correlated with a coefficient of 0.923 [95% confidence interval (CI): 0.905–0.938]. Notably, the fit line fell below the reference line at high bilirubin levels, indicating that Roche TBiL slightly underestimated bilirubin levels relative to Ortho TSB.

**TABLE 1:** Characteristics of the study population.

Characteristic (*n* = 309)	Value
Gestational age (weeks)	38.4 (35.6, 39.7)
Postnatal age (hours)	19.0 (6.8, 45.0)
Male sex	178 (57.6%)
Birth weight (g)	2,811 ± 876
Preterm (<37 weeks)	92/308 (29.9%)
Mode of delivery (*n* = 307)	
Spontaneous vaginal	87/307 (28.3%)
Assisted vaginal	4/307 (1.3%)
Elective cesarean	70/307 (22.8%)
Emergency cesarean	146/307 (47.6%)
Mode of feeding (*n* = 307)	
Breastfeeding only	109/307 (35.5%)
Formula feeding	107/307 (34.9%)
Breastfeeding plus supplement	15/307 (4.9%)
Not feeding at admission	76/307 (24.7%)

To further evaluate the consistency between the two methods, Passing–Bablok regression and Bland–Altman analyses were conducted. The Passing–Bablok regression analysis showed both systematic and proportional differences between the two methods, with a slope of 0.794 (95% CI: 0.763–0.825) and an intercept of 1.255 (95% CI: 1.042–1.417). Figure 1 shows a slight overestimation with the Roche TBiL method at low bilirubin levels, whereas at bilirubin levels greater than 5 mg/dL, Roche TBiL tended to underestimate the levels relative to Ortho TSB in a concentration-dependent manner.

**FIGURE 2:**
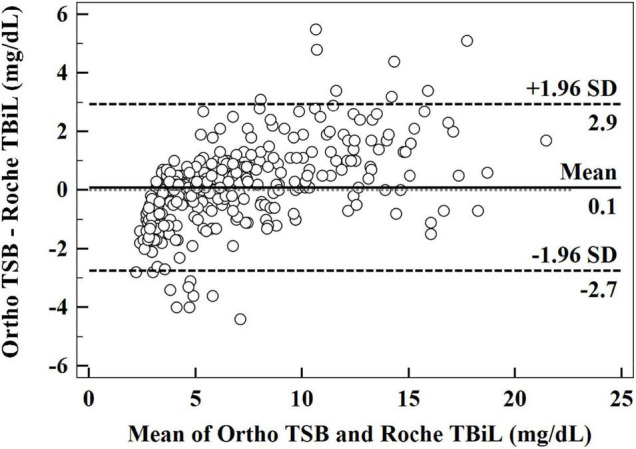
Bland–Altman plot illustrating the relationship between the difference in Ortho TSB and Roche TBiL and the mean of the two sets of data.

The Bland–Altman analysis yielded a small average difference of 0.1 mg/dL between the measurements obtained with the Roche TBiL and Ortho TSB, but with a wide 95% limit of agreement (LoA) range (−2.7 to 2.9 mg/dL). Consistent with the results of the Passing–Bablok regression analysis, the Bland–Altman plot (Figure 2) showed a trend of overestimation with Roche TBiL at low bilirubin levels, with eight paired data points (8/309) falling below the lower 95% LoA. However, a trend of underestimation was significant at high bilirubin levels, with eight paired values (8/309) exceeding the upper 95% LoA.

**FIGURE 3:**
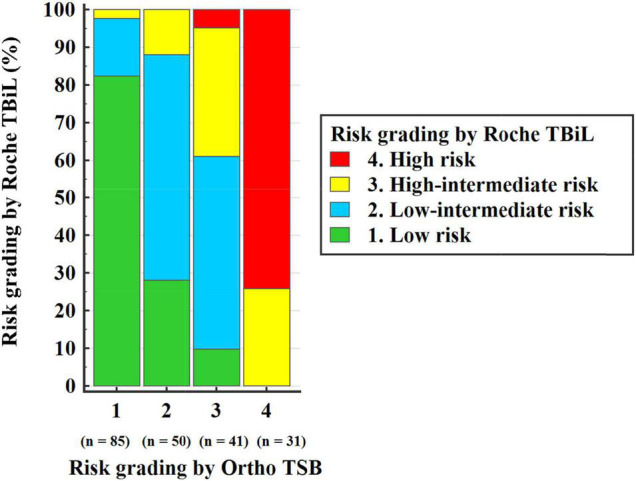
Subsequent neonatal hyperbilirubinemia risk grading by Roche TBiL and Ortho TSB.

We next evaluated the reliability of the Roche TBiL for risk stratification of neonatal hyperbilirubinemia. Among the 309 neonates included in the above measurement accuracy study, 207 term newborns aged between 12 and 144 h were eligible for risk evaluation. Kappa analysis showed good agreement between the Roche TBiL and Ortho TSB in the subsequent hyperbilirubinemia risk evaluation, with a weighted kappa value of 0.681 (95% CI: 0.610–0.751) across all four risk zones. However, as Table 2 and Figure 3 show, the subgroup data indicated substantial errors for Roche TBiL measurements, especially for infants in the intermediate- and high-risk zones (as defined by Ortho TSB). Roche TBiL tending to underestimate the risk of developing severe hyperbilirubinemia. In the low-intermediate risk group, 14 of 50 neonates were underestimated as showing low risk; moreover, approximately half (21 of 41) of the neonates with high-intermediate risk were misclassified into the low-intermediate risk zone. The number of under-or over-estimations beyond these two grades was much lower at 9.8% (4/41) and 4.9% (2/41), respectively.

**TABLE 2:** Kappa analysis of Roche TBiL and Ortho TSB in neonatal hyperbilirubinemia risk grading.

Roche TBiL	Ortho TBS				
	Low (*n* = 85)	Low-intermediate (*n* = 50)	High-intermediate (*n* = 41)	High (*n* = 31)	*n*
Low	**70**	14	4	0	88
Low-intermediate	13	**30**	21	0	64
High-intermediate	2	6	**14**	8	30
High	0	0	2	**23**	25
Weighted kappa (95% CI)		0.681 (0.610∼0.751)			

*The bold values represent the number of cases consistent in risk grading according to Ortho TSB and Roche TBiL measurements. We suggest changing them to a plain style to fulfill publication specifications.*

## Discussion

Our findings suggest that the results obtained with Roche TBiL and Ortho TSB are not consistent in the neonatal population, especially in neonates at a high risk of hyperbilirubinemia. Similar to the results of previous studies comparing whole blood bilirubin measurements with TSB (5, 6), our data showed a good correlation (*r* = 0.923) between Roche TBiL and Ortho TSB measurements. However, the Passing–Bablok regression analysis indicated both systematic and proportional differences between the two methods. In our study, the average difference between Roche TBiL and Ortho TSB measurements was 0.1 ± 1.448 mg/dL across a bilirubin range of 2–25 mg/dL. A recent study reported a more significant bias between whole blood bilirubin measurements using the GEM blood gas analyzer and the VITROS BuBc methodology, with a negative bias at low bilirubin levels and a positive bias at higher levels (7). Interestingly, another study found that the whole blood bilirubin measurements obtained by the Radiometer blood gas analyzer are closely correlated with the measurements obtained using the Roche plasma diazo method, with a mean bias of −1.0 μmol/L and a 95% CI range of −20.00 to 19.00 μmol/L. However, a statistically significant underestimation was identified compared with the VITROS 350 with a mean bias of –4.4 μmol/L and a 95% CI of –29.90 to 21.10 μmol/L (5). Collectively, these and our studies suggest that the accuracy of whole blood bilirubin measurements is instrument-dependent, and local validation is warranted before clinical application, particularly in specific patient populations whose bilirubin levels need routine monitoring.

As shown in the Bland–Altman plot (Figure 2), among our 309 paired measurements, 2.6% (8/309) of Roche TBiL results underestimated TSB levels by more than 3 mg/dL, which is clinically relevant in some situations (8). To further explore the extent to which the difference between the two methods may influence clinical decision-making, we input the two sets of values into the Bhutani nomogram, respectively (4). Statistically, the weighted kappa analysis showed good agreement between Roche TBiL and Ortho TSB for neonatal hyperbilirubinemia risk assessment across all level populations. However, this result may be somewhat limited by the fact that a relatively small number of the high-risk neonates were included in the current study. For newborn infants with high-intermediate risk, Roche TBiL underestimated TSB levels in over half (25/41) of the cases. Moreover, for comparisons at the high-risk zone, which would trigger the initiation of phototherapy, Roche TBiL underestimated measurements in eight of 31 neonates. Similarly, in a prior study including 65 neonates in the high-risk zone, 23 cases were underestimated by whole blood bilirubin measurements (7). In addition to underestimations, overestimations caused by Roche TBiL measurements were also determined in the low-risk and intermediate-risk groups. These results underline the importance of accurate and precise bilirubin measurements in neonatal jaundice management.

Since the first studies evaluating the consistency between whole blood bilirubin and TSB measurements were published in 2001 (9, 10), many factors that may contribute to a bias in whole blood bilirubin measurements have been proposed. First, for plasma soluble substance determination, the sample dilution effect of red blood cells in whole blood samples is an apparent problem that must be properly accounted for. Currently, most blood gas analyzer-based whole blood bilirubin measurements report the plasma-equivalent bilirubin level through a calculation that relies on the measurement of hemoglobin, which is converted to hematocrit to correct for the sample dilution of RBCs (5, 7, 11). This step is of great importance for accurate hemoglobin measurements in this setting. Unfortunately, the reliability of hemoglobin measurement using a blood gas analyzer remains controversial (12, 13). Huang et al. (14) found that hemoglobin measurements using a GEM 4000 blood gas analyzer is consistent with the hematology analyzer results in adults, but a significant bias is using neonatal blood. In line with Huang’s study, an experience from an NICU in Australia demonstrated that blood gas analyzer (Radiometer ABL 800 flex) results for neonatal bilirubin should be corrected according to fetal hemoglobin (HbF) values rather than adult hemoglobin measurements (15). Since the absorbance spectra of adult hemoglobin and HbF are different, the results will differ.

Another critical element that will influence the differences across methods is the calibration procedure. In a study with a significantly large sample size, Kuzniewicz et al. (16) described the consequences of Ortho Diagnostics altering the values assigned to their calibrators for neonatal bilirubin assays. The recalibration resulted in neonate bilirubin concentrations decreasing by 1.18 mg/dL (equal to 20.2 μmol/L) and a 39% relative reduction of infants above the threshold for phototherapy treatment (17). Similarly, in a study investigating the clinical impact of implementation of a new formula provided by Roche Diagnostics for predicting TSB levels, Martha et al. (18) reported a 7% reduction in the number of neonates with results that triggered the initiation of phototherapy treatment. In our study, the Roche TBiL measurement was calibrated to TSB measurements (11), whereas the reference method (VITROS BuBc) was calibrated to measurements of unconjugated and conjugated bilirubin, and the sum was reported as TSB (19). Consequently, the difference between the two methods in the current study can be partially explained by the disparate calibration procedures.

## Conclusion

In conclusion, the results of our study indicate that bilirubin measurements obtained with Roche TBiL and Ortho TSB are not consistent in the neonatal population, especially for those at high risk of developing severe hyperbilirubinemia. As a point-of-care and trace blood assay, Roche TBiL measurement can facilitate neonatal hyperbilirubinemia screening, but it seems to lack accuracy regarding the Bhutani nomogram-based risk stratification, particularly for high-risk newborn individuals at present.

## Data Availability Statement

The datasets generated for this study are available on request to the corresponding author. Requests to access these datasets should be directed to XZ, freedomzxl@163.com.

## Ethics Statement

The studies involving human participants were reviewed and approved by the Ethics Committee of the General Hospital of Tianjin Medical University (no. IRB2022-WZ-011). Written informed consent from the participants’ legal guardian/next of kin was not required to participate in this study in accordance with the national legislation and the institutional requirements.

## Author Contributions

XZ and QW contributed to the conception and study design. QW, TZ, and YL drafted the manuscript. XZ critically revised the manuscript. QW, TZ, YL, LJ, and WZ collected and analyzed the data used in the study. All authors gave final approval and agreed to be accountable for all aspects of work ensuring integrity and accuracy.

## Conflict of Interest

The authors declare that the research was conducted in the absence of any commercial or financial relationships that could be construed as a potential conflict of interest.

## Publisher’s Note

All claims expressed in this article are solely those of the authors and do not necessarily represent those of their affiliated organizations, or those of the publisher, the editors and the reviewers. Any product that may be evaluated in this article, or claim that may be made by its manufacturer, is not guaranteed or endorsed by the publisher.
